# Articulating Concepts Matters! Resilient Actions in the Norwegian Governmental Response to the COVID-19 Pandemic: Comment on "Government Actions and Their Relation to Resilience in Healthcare During the COVID-19 Pandemic in New South Wales, Australia and Ontario, Canada"

**DOI:** 10.34172/ijhpm.2022.6892

**Published:** 2022-02-05

**Authors:** Sina Furnes Øyri, Siri Wiig

**Affiliations:** Faculty of Health Sciences, SHARE - Centre for Resilience in Healthcare, University of Stavanger, Stavanger, Norway.

**Keywords:** COVID-19 Pandemic, Norwegian Governmental Response, Situated Resilience, Structural Resilience, Systemic Resilience

## Abstract

The coronavirus disease 2019 (COVID-19) pandemic has challenged our healthcare systems and required collaboration from both centralized and decentralized system levels to adapt to the changes and challenges. This commentary offers a look into the Norwegian governmental healthcare system and response within a resilience in healthcare perspective, by analyzing the situated, structural, and systemic resilience. Such a conceptualization of resilience into three scales of organizational activity may assist our efforts to *understand* and *explain* governmental actions throughout the pandemic. Research application of resilience in healthcare to explain and discuss government actions during the COVID-19 pandemic, needs to ensure sensitivity to the overall structural, cultural, and human factor aspects of the relevant healthcare system under scrutiny as well as sensitivity to specific context *within* the various system levels.

## Background

 The coronavirus disease 2019 (COVID-19) pandemic has challenged every healthcare system across the globe. Any government action has depended on localized will, competences, and resources to understand, adapt and respond to the changes the pandemic has required.^1^ The implications of successful plans and actions initiated by a centralized government have been closely linked with decentralized actions from the public, health leaders, and healthcare personnel in the entire healthcare system. This duality of required collaboration from both centralized and decentralized system levels, resonates with the resilience in healthcare concept.^2^

 Smaggus and colleagues’ study of “Government Actions and Their Relation to Resilience in Healthcare During the COVID-19 Pandemic in New South Wales, Australia and Ontario, Canada,”^3^ provides a highly interesting look into how governmental actions in two jurisdictions during the COVID-19 pandemic related to the concept of resilience. Their key message is that government actions addressed resilience potentials, although “resilience” was not always explicitly applied in the media releases. Smaggus and colleagues address how the articulation of resilience in government actions may account for an important organizing principle across system levels in the healthcare setting. Investigating the governmental level with a resilience perspective, as done by Smaggus and colleagues is novel, needed, and challenging. In this paper we seek to constructively comment on this based on Norwegian policy documents issued throughout the pandemic and how we see this linked to situated, structural and systemic resilience.

###  The Norwegian Health System

 The Norwegian society as a whole and the healthcare system specifically, is funded on principles of equality, equity, and power distribution.^4^ The power structures are highly distributed (see Box 1).

**Box 1. Power Distribution in the Norwegian Healthcare System**

**National; Centralized System Level**

- The central Government body in the healthcare setting is led by the Ministry of Health and Care Services (the Ministry).^5^
- The Ministry directs the Norwegian healthcare services through comprehensive legislation, annual budgetary allocations and by means of various governmental institutions such as the Norwegian Directorate of Health (the Directorate) and the Norwegian Institute of Public Health (the Institute).^5^

**Regional and Local; Decentralized System Levels**

- The decentralized levels of power relate to regional and local authorities in the municipalities across the country.
- The municipalities are responsible for primary care in terms of nursing homes, homecare, ambulatory care, and general practitioners.^5^
- Four regional health authorities are set to implement the national health policies, and to plan, organize, govern, and coordinate all subordinated local health trusts (hospitals) in their region (the Health Trusts’ Act of 2001).^6^

The national healthcare contingency plan issued by the Ministry in 2018 clarifies the overall framework for the healthcare system’s preventive measures and risk management in cases of crisis, and disasters.^7^ It holds national requirements and regional and local recommendations for measures, based on experiences retrieved from the Ebola outbreak in West Africa 2014-2015 and the plan thus forms the governmental response and actions during the COVID-19 pandemic.^7^ The Government presupposes agreement among different stakeholders about the key principles in emergency preparedness, as being related to a set of basic elements: get knowledge about and gain overview of risk and vulnerabilities, put preventive measures in place to prevent adverse events and crisis, have sufficient contingency to deal with adverse events and crisis, restore functions during and after adverse events, crisis, and disasters, and learn from experiences retrieved from adverse events and simulation.^7^

###  Resilience as an Unarticulated Integrated Concept

 The national contingency plan does not explicitly refer to resilience, even though several of the basic elements echo the resilience in healthcare concept.^7^ Despite the elaborate contingency plan, with attention to the various responsibilities at central, regional, and local municipality levels, the Norwegian governmental role and response during the pandemic has been extensively debated, with different views about the balance between centralized control and the degree of regional autonomy within the municipalities.^8^

 Since the term “resilience” does not have a proper Norwegian translation, we did not expect and did not find the term in the two main Norwegian governmental documents that we base our commentary on:

- the white paper “Meld. St. 11 (2020-2021) Quality and patient safety 2019” issued by the Ministry.^9^
- the report “NOU 2021:6 The authorities’ handling of the COVID-19 pandemic,” provided by the Coronavirus Commission.^4^

In contrast to the documents issued in Norwegian, the English summary of the white paper “NOU 2021: 6,” includes three references to “a resilient society.” The lack of “resilience” terminology was however remedied by deductively searching for the resilience potentials of adaptive capacity, anticipation, learning, monitoring, and responding. Similarly, Smaggus and colleagues, did neither find exact definitions of “resilience.”^3^ However, as noted by Smaggus and colleagues, the absence of precise definitions of resilience in government actions does not hinder *elements of resilience* being evident in the same actions or documentary evidence.^3^ The latter aligns with our take home message as well. Looking at the definition provided by the ongoing Resilience in Healthcare Research Program (2018-2023), resilience is defined as “the capacity to adapt to challenges and changes at different system levels, to maintain high quality care.”^10^ In the documentary evidence we have assessed, we found one of the Norwegian government’s responses to echo the core principle of this definition, without referring to the term resilience itself:

 “Due to the pandemic, hospitals have had to strengthen their preparedness to treat patients with COVID-19 in addition to other patient groups. During a period when the situation was uncertain, the hospitals had to reduce the normal activities to ensure adequate preparedness for handling patients with COVID-19 and to prevent possible spread of infection. As a result, many patients had their assessment or treatment postponed.”^9^ Thus, as the quotation illustrates, the Government describes key attributes of the resilience in healthcare concept in their actions despite the absence of the term “resilience” in government actions. Our finding corresponds to the analysis of Smaggus and colleagues.

###  Situated, Structural, Systemic Resilience 

 This comment addresses the Norwegian government actions related to resilience in healthcare by analyzing the situated, structural, and systemic resilience, as this conceptualization of resilience into three scales of organizational activity may assist our efforts to *understand *and *explain* governmental actions throughout the pandemic. The three scales of situated, structural, and systemic resilience address processes of activities and changes across system levels.^11^ In theory, situated resilience relates to readjustment of practices and activities at the micro level, structural resilience links with processes of reorganizing and restructuring of operational activities and resources often found at the monitoring meso level, whilst systemic resilience emerges in the reconfiguring or reformation of resource design and production, often found at the overseeing, macro level of the system.^11^

 Characterized as situated resilience, available resources in the services needed restructuring to deal with the new risks displayed by COVID-19, with managerial responsibilities as important facilitators for change both in hospitals and in nursing homes. More specifically, situated readjustment at the micro hospital level was found in readjustment of activities as indicated by a reduction in activity overall, a decrease of physical consultations, changes in numbers for cancer patient pathways as well as increased waiting time for medical investigations, treatment and follow-up in March and April of 2020 with deadlines not being met.^9^ These readjustments may have some unfortunate long-term consequences which must be evaluated.^9^ The pandemic has however led to positive innovations as well, displaying structural reorganizing and restructuring through increased use of digital tools such as video consultations to maintain important services during the pandemic.^9^ Structural changes were also found in the establishment of different types of arenas for testing, and systems related to ensure access control for patients and visitors to hospitals and nursing homes.^9^ Changes at the administrative and managerial levels in healthcare organizations were recently reflected in findings from a study about healthcare leaders’ use of innovative solutions to ensure resilience in healthcare during the COVID-19 pandemic.^12^ The study notes that innovation is a central aspect to adaptive capacity and demonstrates that already existing technology was adapted to new settings, and physical innovations were for instance found in the establishment of “infection and non-infection wards” and “protective equipment changing areas.”^12^ Restructuring of resources and innovative solutions have indeed been paramount. However, some groups have gained a heavier workload compared to a normal state prior to the pandemic. This may have resulted in some negative implications: resource pressure and heavier workload may contribute to a potential increase of undeliberate mistakes, especially in cases of handling medication.^9^ In addition, and as explicated in a recent article by Riess,^13^ governments and healthcare organizations must keep attention to human factors at an individual level such as (1) Safety; (2) Access to information that is Accurate and Caring; (3) Maintenance of Human Connection; (4) Emphasis on Mental Health, to reduce the current crisis’ negative implications to every level of the system. References to these factors are absent in Smaggus’ article. Individual resilience to cope with resource pressure and heavier workload, is dependent on higher level structures. We therefore urge the governments in charge of the healthcare systems, to learn from the experiences affiliated with local level safety, communication, community, and mental health needs, to ensure societal and organizational resilience in future crisis and pandemics.

 We found systemic reformation at the macro level by means of restructuring the entire health service, to prevent infection, control the spread of and treatment of the virus. Overall, the fact that all parts of the service had to deal with changes and coordinate resources across units, departments and clinics testifies to resilience seen as a system wide capacity. Especially health personnel have mobilized extraordinarily and made prudent changes to meet with disruptions and constant shifts in circumstances, demonstrating localized adaptations. During the first phase of the pandemic, measures needed quick implementation, with plans for further work being established under considerable uncertainty, related both to the potential consequences of the virus and the development of the pandemic in general.^9^ At the same time, ordinary tasks had to be handled as best possible. This combination required both the application of already established contingency plans and the ability to adapt, while at the same time maintaining operations. Moreover, systemic resilience was demonstrated in the Norwegian government’s expectations of increased attention to technological and digital solutions. Digital collaboration among system levels was key to get information across and reach the public. In addition, video consultations between healthcare personnel and patients were launched as part of a reconfiguring process to uphold essential parts of the services. The Government defines these reconfigurations as important contributions to maintain the societal emergency preparedness, as well as take home messages for future development of the services.^9^ Likewise, the work of Smaggus and colleagues’ original article, demonstrates links between the importance of facilitating planning of resource reorganization (ref. “existing actions”), and attention to learning in how to identify system boundaries (ref. “the more proactive forms of resilience”).^3^ In our view, the latter shows how the resilience potential of adaptive capacity may fuse with the resilience potential of learning. Moreover, as Smaggus and colleagues’ note in their article: “media releases also revealed opportunities to enhance learning (eg a need to capitalize on opportunities for double-loop learning and identify strategies appropriate for complex systems) and anticipating.”^3^ The take home message it displays, is that learning from past response to this type of crisis, may be used to better arm the systems’ anticipatory capacities. By looking at the outputs from the (still ongoing) COVID-19 pandemic, we find that the intertwined potentials of adaptive capacity, anticipation and learning may be helpful to explicate our efforts to ensure a more resilient responding system to the unknown future that lies ahead of us.

 Relating all three scales of situated, structural, and systemic resilience to findings from the governmental documents investigated in our commentary, it implies that the Norwegian government expects a systemic process to occur as result from their actions during the COVID-19 pandemic.^11^ Thus, as actions involved deep collaboration among all system levels and among all stakeholders involved at each level, regional and local adaptive capacity contributed to the potential for resilient performance in our health system, potentially serving us well in terms of implementation of and effect of control measures. We agree with Smaggus and colleagues^3^ that it is key to stronger articulate the importance of resilience in healthcare and the themes, potentials, and mechanisms it depends on. The urgency of doing this at the macro level, is shown here as a pandemic response that needs national anchoring and system wide actions.

###  “New Challenges and Opportunities” – Consequences of Situated, Structural, and Systemic Resilience

 The Norwegian government stresses how postponed assessment or patient treatment to some extent will have consequences for the entire population, but first and foremost represents new challenges to vulnerable patient groups and next of kin.^9^ We see that the relation between pandemic response and resilience has conflicting potential. From an organizational and societal perspective, both small scale and large-scale adaptations are key to handle the crisis, but at an individual level, we do not know the long-term consequences.^1^ From a governmental point of view, the entire system responded by reinventing itself at different levels, but the tangible adaptations may result in unfortunate consequences due to postponed examinations, delayed clinical decision-making and treatment. The latter shows how structural and situated adaptations during the pandemic may have resulted in non-beneficial outcomes for patients. Forthcoming statistics will contribute to reveal these types of long-term consequences and will form a useful learning platform in future systemic reformation processes.^4,9^ In parallel, as Smaggus and colleagues’ note, part of the governments responses related to “new challenges and opportunities” caused by the pandemic, for instance resulting in the introduction of “programs to address mental health challenges,” both in New South Wales and Ontario.^3^ Interestingly, new challenges may in that sense lead to potentially unfortunate consequences in a patient safety perspective. On the other hand, it may also represent new opportunities in a perspective of quality improvement, when the governments ought to facilitate special measures to remedy the potential risks affiliated with an ongoing crisis. Readjustment of practices and activities at a local level thus may lead to reorganizing and restructuring of operational activities and resources, in line with different scales of resilience.^11^

###  Sensitivity to Different System Contexts in the Application of Resilience in Healthcare

 The pandemic has not only proven to be a health crisis with individual impact but with extensive additional impact on the system performance overall. Comparisons across different countries, however, indicate that cultural and structural aspects impact the spread of the virus and the efficiency of governmental actions.^14^ In a Norwegian context, the Norwegian Government actions, and measures to control the pandemic have in an international perspective been modest and “less intrusive” compared to most other high-income countries.^9^ This contrasting fact has interesting implications to how we conclude our comment on Smaggus and colleagues’ examination of government actions and their relation to resilience in healthcare. Several “distinctive features” of the Norwegian society structures and embedded values may have had significant impact on the healthcare systems’ ability to be resilient throughout the pandemic.^4^ The Coronavirus Commission refers to trust and solidarity, the Nordic model, a well-developed health system, digitalization, and the ability to work from home, adaptability, and effort.^4^ These features played vital roles in shaping the authorities’ adaptive capacity in their quick response and decision making. Moreover, the authorities relied heavily on the population’s trust in the measures, whilst being able to react and adapt quickly. We believe that this reflects the combined effort of macro level emergency preparedness with the phase of monitoring the spreading of the virus, and anticipating the infection’s risk potential, and the responses made to adhere the risks, in turn with implications for the meso (population) and micro (hospitals) levels’ response to the measures. Our commentary notes that this chain of activities was redeemed by resilient capacities found at different system levels in the Norwegian health system^1,2,4,7,9^ and may add to the explanation of why the Norwegian system has experienced low mortality rates. It moreover implies that research application of resilience in healthcare to explain and discuss government actions during the COVID-19 pandemic, needs to ensure sensitivity to the overall structural and cultural aspects of the relevant healthcare system under scrutiny as well as sensitivity to specific context *within *the various system levels. To conclude, articulating resilience with a strong link to context sensitivity and governmental role and action appears crucial to support leveraging resilience into practice as a multilevel phenomenon in any healthcare system.

## Acknowledgements

 We extend our thanks to the Editor-in-Chief for inviting us to write this commentary. We would also like to thank the referee for valuable comments.

## Ethical issues

 Not applicable.

## Competing interests

 Authors declare that they have no competing interests.

## Authors’ contributions

 SFO collected and analysed the documents and drafted the manuscript. SW contributed substantially with comments and revisions. Both authors made critical revisions to the manuscript’s scientific content.
